# Using extended concentration and achievement indices to study socioeconomic inequality in chronic childhood malnutrition: the case of Nigeria

**DOI:** 10.1186/1475-9276-8-22

**Published:** 2009-06-05

**Authors:** Olalekan A Uthman

**Affiliations:** 1 Center for Evidence-Based Global Health, Ilorin, PO Box 5146, Kwara State, Nigeria; 2 Department of Public Health and Epidemiology, University of Birmingham, Edgbaston, Birmingham, B15 2TT, UK

## Abstract

**Objectives:**

To assess and quantify the magnitude of inequalities in under-five child malnutrition, particularly those ascribable to socio-economic status

**Methods:**

Data on 4187 under-five children were derived from the Nigeria 2003 Demographic and Health Survey. Household asset index was used as the main indicator of socio-economic status. Socio-economic inequality in chronic childhood malnutrition was measured using the "extended" illness concentration and achievement indices.

**Results:**

There are considerable pro-rich inequalities in the distribution of stunting. South-east and south-west regions had low average levels of childhood malnutrition, but the inequalities between the poor and the better-off were very large. By contrast, North-east and North-west had fairly small gaps between the poor and the better-off on childhood malnutrition, but the average values of the childhood malnutrition was extremely high.

**Conclusion:**

There are significant differences in under-five child malnutrition that favour the better-off of society as a whole and all geopolitical regions. Like other studies have reported, reliance on global averages alone can be misleading. Thus there is a need for evaluating policies not only in terms of improvements in averages, but also improvements in distribution.

## Background

More than one-quarter of all under fives in the developing world are underweight [1]. This accounts for about 143 million underweight children in developing countries [1]. Of these 143 million underweight children, nearly three-quarters live in just 10 countries [1]. In Sub-Saharan Africa more than one-quarter of children under five are underweight. Nigeria and Ethiopia alone account for more than one-third of all underweight children in Sub-Saharan Africa [1]. Undernutrition, conversely, has been estimated to be an underlying cause for around half of all child deaths worldwide [2]. According to recent comparative risk assessments, under-nutrition is estimated to be, by far, the largest contributor to the global burden of disease [2,3]. Undernourished children have lowered resistance to infection and are more likely to die from common childhood ailments like diarrhoeal disease and respiratory infection. Frequent illness saps the nutritional status of those who survive, locking them into a vicious cycle of recurring sickness and faltering growth. Their plight is largely invisible: Three quarters of the children who die from causes related to malnutrition were only mildly or moderately undernourished, showing no outward sign of their vulnerability [1].

The Millennium Development Goals (MDGs) state as the first goal "to halve between 1990 and 2015 the proportion of people who suffer from hunger." One indicator to monitor progress for this target is the proportion of children who are underweight – i.e. low weight compared with that expected for a well-nourished child of that age and sex. Child malnutrition is one of the measures of health status that the World Health Organization (WHO) recommends for equity in health [4]. From the existing evidence it is clear that childhood malnutrition is associated with a number of socioeconomic and environmental characteristics such as poverty, parent's education/occupation, sanitation, rural/urban residence and access to health care services. Also demographic factors such as the child's age and sex, birth interval and mother's age at birth have been linked with malnutrition [5-8]. In addition, previous studies have drawn attention to the disproportional burden of malnutrition among children from poor households [4,5]. There seems to be a broad agreement that many socioeconomic inequalities are unfair [9], because they are result of a division of labour in society that puts certain groups of people at a disadvantage, not only economically, socially, and politically but also in terms of their possibilities to be healthy [10]. Inequalities in health arise, in part, because of inequalities in society [11]. There is no society without inequalities [11]. It is a major challenge to reduce the magnitude of social inequalities in health. To do so requires commitment and concerted action across many sectors of society.

In the biomedical field, linear and logistic regression analyses are the classical approaches to studying the association between socioeconomic position (SEP) and childhood malnutrition [12]. Usually, odds ratios (OR) or beta coefficients are reported to indicate the magnitude and direction of the association [13,14]. These methods are straightforward, but suffer from several limitations. First, although traditional regression analysis can help examine whether there is an association between SEP and childhood malnutrition, it is not powerful enough to measure the disparity quantitatively, i.e., to tell how severe the inequality is. Second, comparing inequality across studies or over time using traditional regression analysis is difficult, since the validity of regression analysis is based on the assumption of multi-normality and independence between study variables over time [15]. Third, from a statistical perspective, linear regression analysis assesses the relationship between the outcome and explanatory variables *on average *but ignores the possibility that the effect of explanatory variables may vary across the distribution. To solve similar problems, economists have developed summary indices such as the Gini coefficient and the concentration index to quantitatively measure the degree of income-related inequality [16]. Unlike Gini coefficient, the concentration index meets all three important criteria that a good measure of inequality is expected to fulfill [17]: (1) it takes account of the socio-economic dimension of inequality in health; (2) it reflects the experience of the entire population rather than two extreme groups on the socio-economic scale (e.g. income quintile 5 versus income quintile 1) as is the case in range measures (e.g. rate-ratios), and (3) it is sensitive to changes in the population across socio-economic groups. The concentration index has proven as a useful tool for measuring inequalities in the health sector [18] and have been used extensively in public health to studies socioeconomic inequality in self-rated health [19,17], child injury [20], ownership of insecticide net [21-23], measles immunization coverage [24], childhood malnutrition [4,25-27], overweight [28], obesity [12], mental health [11], and infant mortality [29,30].

Despite these strengths, it does however have limitations [31]. First, like the Gini coefficient, it has implicit in it a particular set of value judgments about aversion to inequality. However, the "extended" concentration index proposed by Wagstaff's [32] allows attitudes to inequality to be made explicit, and to see how inequality changes measured as the attitude to inequality changes. The second drawback of the concentration index – and the generalization of it – is that it is just a measure of inequality. Although equity is an important goal of health policy, it is not the only one. It is not just health inequality that matters; the average level of health also matters. Policy makers are likely to be willing to trade one off against the other – a little more inequality might be considered acceptable if the average increases substantially. This point to a second extension of the concentration index [32]: a general measure of health "achievement" that captures inequality in the distribution of health (or some other health sector variable) as well as its mean.

In country like Nigeria with high degree of socioeconomic inequality, the existence of morbidity and mortality differentials related socioeconomic status is not unexpected. However, policies aimed at reducing inequalities, magnitude and determinants of the problem, as policy decisions based on intuition are likely to be misguided [4,33]. To date, few studies have been done to examine differences in childhood malnutrition rates across socioeconomic groups in Nigeria [25,26]. The aim of this paper was therefore, to contribute to the efforts to quantify inequalities in health in Nigeria, by assessing the magnitude of inequalities in malnutrition of under-five children that are ascribable to socio-economic status.

## Methods

### Data source

Analysis of data in this study was based on 4187 children aged 0–59 month(s) included in Nigeria Demographic and Health Survey (NDHS) in 2003. The NDHS collected demographic, socio-economic, and health data from nationally-representative sample of 7620 women aged 15–49 years in 7864 households included in the survey. The state was stratified into 36 states and the Federal Capital Territory (FCT) of Abuja within the six geopolitical regions. Each domain is made up of enumeration areas (EAs) established by a general population and housing census in 1991. The sampling frame was a list of all EAs (clusters). Within each domain, a two-stage sample was selected. The first stage involved selecting 466 clusters (primary sampling units) with a probability proportional to the size, the size being the number of households in the cluster. The second stage involved the systematic sampling of households from the selected clusters.

### Measurement of malnutrition

Nutritional status was measured by height-for-age z-scores (HAZ). A HAZ is the difference between the height of a child and the median height of a child of the same age and sex in a well-nourished reference population divided by the standard deviation in the reference population. The US National Center for Health Statistics (NCHS) reference population is used as reference population [34]. Generally, children whose HAZ is below minus two standard deviations of the median of the reference population are considered chronically malnourished or stunted. The focus of the study is on inequalities in stunting (low height-for-age), measured as negative of the child's HAZ. As stated by Wagstaff et al. [35], there are two reasons for favouring the z-score over a binary variable indicating whether or not the child in question was stunted (i.e. two standard deviations or more below the NCHS mean). First, it conveys information on the depth of malnutrition rather than simply whether or not a child was malnourished. Second, it is amenable to linear regression analysis. The *negative *of the z-score was used to make malnutrition variable easier to interpret – positive values mean increasing in malnutrition.

### Measurement of socioeconomic status

An index of economic status for each household was constructed using principal components analysis [36] based on data from 7864 households. The following variables were used in principal components analysis: number of rooms per capita, having a car, having a motorcycle, having a bicycle, having a fridge, having a television, having a telephone, and kind of heating device. From this the population economic status quintiles were calculated and used in the subsequent modelling.

### Measurement of socioeconomic inequalities in childhood malnutrition

Inequality in childhood malnutrition was measured using the illness concentration, extended concentration, and achievement indices.

### The extended concentration index

The regular concentration index (*C*) is equal to [37]

(1)

where *n *is the sample size, *h*_*i *_is the ill-health indicator for person *i*, μ is the mean level of ill health, and *R*_*i *_is the fractional rank in the living-standards distribution of the *i*th person. The value judgments implicit in *C *are seen most easily when *C *is rewritten in an equivalent way as

(2)

The quantity *h*_*i*_/*n*.μ is the share of health (or ill health) enjoyed (or suffered) by person *i*. This is then weighted in the summation by twice the complement of the person's fractional rank, that is, 2(1 - *R*_*i*_). So, the poorest person has his or her health share weighted by a number close to two. The weights decline in a stepwise fashion, reaching a number close to zero for the richest person. The *C *is simply one minus the sum of these weighted health shares. The range of *C *is from -1 to +1. Negative values imply that malnutrition is more concentrated among poorer children, the opposite with positive values. If all children, irrespective of their socioeconomic status, suffer equally from malnutrition, the *C *would equal zero.

The extended concentration index was computed by means of a convenient regression [31,32]:

(3)

In equation 3, ν is the inequality-aversion parameter, which will be explained below.

The weight attached to the *i*th person's health share, *h*_*i*_/*n*.μ, is now equal to ν (1 - *R*_*i*_) (ν-1), rather than by 2(1 - *R*_*i*_). When ν = 2, the weight is the same as in the regular concentration index; so *C*(2) is the standard concentration index. By contrast, when ν = 1, everyone's health is weighted equally. This is the case in which the value judgment is that inequalities in health do not matter. So, *C*(1) = 0 however unequally health is distributed across the income distribution. As ν is raised above 1, the weight attached to the health of a very poor person rises, and the weight attached to the health of people who are above the 55th percentile decreases. For ν = 6, the weight attached to the health of persons in the top two quintiles is virtually zero. When ν is raised to 8, the weight attached to the health of those in the top *half *of the income distribution is virtually zero.

### Achievement index

The measure of "achievement" proposed in Wagstaff [32] reflects the average level of health and the inequality in health between the poor and the better-off. It is defined as a weighted average of the health levels of the various people in the sample, in which higher weights are attached to poorer people than to better-off people. Thus achievement might be measured by the index:

(4)

This index can be shown to be equal to the following [31,32]:

(5)

When *h *is a measure of ill health (so high values of *I *(ν) are considered bad) and *C *(ν) < 0 (ill health is higher among the poor), inequality serves to raise the value of *I *(ν) above the mean, making achievement worse than it would appear if one were to look just at the mean. If ill-health declines monotonically with income, the greater the degree of inequality aversion, the greater the wedge between the mean, μ, and the value of the index *I *(ν).

The "extended" concentration and achievement indices were computed for all children in the sample, as well as for certain disaggregated categories including maternal education attainment, ethnic group, area of residence (urban areas and rural areas), and six different geopolitical regions. The bootstrap resampling technique was used to calculate the extended concentrated index and achievement index with their standard error, using 500 bootstrap samples. The DHS stratification and the unequal sampling weights as well as household clustering effects were taken into account in the analysis to correct standard errors. All tests were two tailed and *p *< 0.05 was considered significant. Stata 10.2 (Stata Corp, College Station, TX, USA) software was used for analysis.

### Ethics

The study is based on secondary analysis of existing survey data with all identifying information removed. The survey obtained informed consent from mothers of children included in the study before asking any questions and before taking anthropometric measurements.

## Results

**Figure 1:**
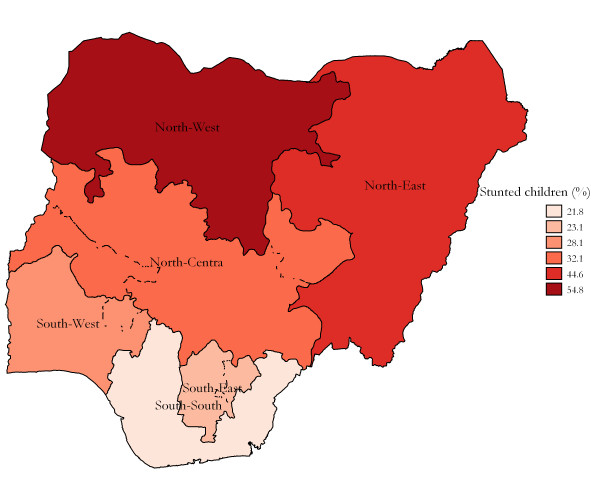
**Estimated prevalence of childhood malnutrition, by geopolitical regions, Nigeria 2003**.

Table 1 shows the childhood malnutrition for different socioeconomic quintiles at national level. It also shows the odds ratio for infant mortality rate and 95% confidence interval for the different quintiles, with the richest quintile being used as the reference category. There is a descending trend in childhood malnutrition rate, the lower rates move up the socioeconomic quintiles. Figure 1 illustrates the rates of children malnutrition by geopolitical regions, as one moves up the north of Nigeria, a remarkable increase in proportion of stunted children was observed. The concentration index indicating socioeconomic inequality of childhood malnutrition in Nigeria was -0.147 (95% confidence interval; -231 to -0.049).

**Table 1:** Estimated childhood malnutrition rate, its odds ratio and 95% confidence interval in socioeconomic quintiles, Nigeria 2003

**Wealth index**	**Mean Negative HAZ**	OR (95% CI)*
Poorest	1.89	3.69 (2.98, 4.58)
Poor	1.81	3.10 (2.50, 3.86)
Rich	1.72	2.80 (2.25, 3.48)
Richer	1.28	1.60 (1.28, 2.00)
Richest	0.82	reference

HAZ: Height-for-age z score; OR: odds ratio; CI: confidence interval

*All estimates are significant at p < .001

### Inequality aversion – extended concentration index

To gain further insight into the nature of the inequality in childhood malnutrition, extended concentration were disintegrated as shown in Table 2. Inequalities to the disadvantage of the poor are evident in all the disintegrated groups. They were especially pronounced South south and South-west, where the value of *C*(2) was equal to -0.251 and -0.288 respectively. In urban areas, the poorest bear the heaviest burden of malnutrition compared to rural areas (C(2) = -0.180 vs C(2) = -0.087). Among the ethnic groups, the pro-rich inequality was more pronounced among the Yorubas and Igbos. The pro-rich inequality was fairly same

**Table 2:** Extended concentration indices and standard errors of childhood malnutrition by maternal education, regions, ethnicity, type of residence, and sex of infant, Nigeria 2003

	**Extended Concentration Index**			
	
**Variable**	***C [SE] (2)***	***C [SE] (3)***	***C [SE] (4)***	***C [SE](5)***
**Maternal education**				
No education	-0.036(0.016)***	-0.0441(0.025)	-0.048 (0.032)	-0.051(0.036)
Primary education	-0.093(0.022)***	-0.132(0.036)***	-0.150 (0.049)**	-0.160(0.051)**
Secondary or higher	-0.200(0.028)***	-0.287(0.046)***	-0.348(0.057)***	-0.395(0.072)***
**Region**				
North central	-0.133(0.030)***	-0.187(0.042)***	-0.212(0.051)***	-0.223(0.061)***
North east	-0.102(0.024)***	-0.142(0.040)***	-0.165(0.050)**	-0.181(0.060)**
North west	-0.065(0.017)***	-0.080(0.029)**	-0.089(0.035)*	-0.097(0.039)*
South east	-0.191(0.039)***	-0.332(0.061)***	-0.446(0.080)***	-0.538(0.099)***
South south	-0.251(0.053)***	-0.349(0.090)***	-0.391(0.118)**	-0.411(0.146)**
South west	-0.288(0.039)***	-0.427(0.067)***	-0.516(0.094)***	-0.579(0.106)***
**Type of residence**				
Urban	-0.180(0.021)***	-0.267(0.038)***	-0.326(0.051)***	-0.373(0.057)***
Rural	-0.087(0.018)***	-0.108(0.027)***	-0.117(0.033)**	-0.121(0.038)**
**Ethnicity**				
Hausa/Fulani	-0.047 (0.015)**	-0.062 (0.021)**	-0.071(0.030)*	-0.079(0.034)*
Igbo	-0.275(0.038)***	-0.465(0.059)***	-0.607(0.072)***	-0.719(0.092)***
Yoruba	-0.222(0.041)***	-0.326(0.065)***	-0.399(0.088)***	-0.459(0.105)***
Others	-0.162(0.020)***	-0.220(0.034)***	-0.251(0.044)***	-0.272(0.057)***
**Child's sex**				
Male	-0.146(0.016)***	-0.200(0.026)***	-0.227(0.033)***	-0.242(0.038)***
Female	-0.148(0.017)***	-0.197(0.028)***	-0.219(0.038)***	-0.230(0.044)***
**Overall**	-0.147(0.013)***	-0.199(0.022)***	-0.223(0.028)***	-0.236(0.034)***

The * for p < .05, ** for p < .01, and *** for p < .001 (based upon bootstrapped standard errors).

Raising the value of *ν *above 2 results in increasing pro-rich inequality. Thus, for all children studied in Nigeria in 2003, average value of *C*(5) is -0.236 whilst the average value of *C*(2) was only -0.147. Interestingly, the impact of raising *ν *varies sub-groups studied. For example, raising the value of *ν *from 2 to 5 causes the extended concentration index for malnutrition in South-east to fall from -0.191 to -0.538 – a three-fold change. By contrast in North-west, the change was far smaller – from -0.065 to -0.097. This reflects the fact that in South-east, the inequality amongst the poorest group differs quite dramatically from the rest of the sample. Another group whose extended concentration index was highly sensitive to the choice of *ν *was ethnicity. Raising the value of *ν *from 2 to 5 for Igbo causes the extended concentration index to fall from -0.275 to -0.719. This reflects the fact that the malnutrition amongst the poorest quintile among Igbos was much higher than that amongst the other four quintiles. Similarly, socioeconomic inequalities varied according to maternal education attainment. The magnitude of socioeconomic inequality in childhood malnutrition increases with increasing maternal education. However, impact of raising *v *from 2 to 5 was similar across maternal levels of education.

### Achievement – trading off inequality and the mean

Table 3 shows achievement indices for childhood malnutrition for overall children and according to maternal education, region, sex, type of residence and ethnicity. Focus on the achievement index produces some interesting results, especially for the regions and ethnicity. In general, raising values of *I *from 1 to 5 the achievement indices rises further and further above the mean, meaning that the level of "disachievement" becomes larger and larger. This "disachievement" is more pronounced in North-west and among Hausas and Fulanis.

**Table 3:** Achievement indices and standard errors of childhood malnutrition by maternal education, regions, ethnicity, type of residence, and sex of infant, Nigeria 2003

***Characteristics***	**Achievement Index (I)**				
	
	I(1) [SE]*	I(2) [SE]*	I(3) [SE]*	I(4) [SE]*	I(5) [SE]*
**Maternal education**					
No education	1.905	1.973 (0.031)	1.989 (0.047)	2.000 (0.062)	2.002 (0.069)
Primary education	1.464	1.602 (0.033)	1.658 (0.053)	1.685 (0.071)	1.698 (0.074)
Secondary or higher	0.904	1.083 (0.025)	1.164 (0.042)	1.219 (0.051)	1.261 (0.065)
**Region**					
North central	1.234	1.399 (0.037)	1.466 (0.051)	1.496 (0.063)	1.510 (0.075)
North east	1.709	1.882 (0.041)	1.951 (0.067)	1.990 (0.086)	2.018 (0.103)
North west	2.131	2.269 (0.037)	2.303 (0.062)	2.321 (0.074)	2.338 (0.083)
South east	0.984	1.171 (0.039)	1.310 (0.060)	1.422 (0.079)	1.513 (0.097)
South south	0.854	1.068 (0.046)	1.152 (0.077)	1.188 (0.100)	1.205 (0.124)
South west	1.084	1.396 (0.042)	1.547 (0.073)	1.643 (0.102)	1.712 (0.115)
**Type of residence**					
Urban	1.214	1.433 (0.026)	1.538 (0.047)	1.610 (0.062)	1.667 (0.070)
Rural	1.696	1.844 (0.030)	1.880 (0.046)	1.894 (0.057)	1.902 (0.064)
**Ethnicity**					
Hausa/Fulani	2.085	2.183 (0.031)	2.215 (0.043)	2.234 (0.062)	2.251 (0.070)
Igbo	0.851	1.086 (0.032)	1.247 (0.051)	1.370 (0.061)	1.464 (0.079)
Yoruba	1.135	1.387 (0.047)	1.505 (0.073)	1.587 (0.100)	1.655 (0.119)
Others	1.309	1.521 (0.026)	1.597 (0.044)	1.637 (0.058)	1.665 (0.074)
**Child's sex**					
Male	1.570	1.799 (0.016)	1.884 (0.041)	1.926 (0.052)	1.950 (0.059)
Female	1.453	1.668 (0.024)	1.740 (0.041)	1.771 (0.055)	1.787 (0.064)
**Overall**	1.512	1.734 (0.021)	1.813 (0.034)	1.850 (0.042)	1.869 (0.051)

*All estimates are significant at p < .001 (based upon bootstrapped standard errors).

## Discussion

This study based on nationally representative cross-section of Nigeria, found that there is evidence of concentration of childhood malnutrition and increasing "disachievement" in childhood malnutrition among the poorest. Socioeconomic inequality in childhood malnutrition found in this study is similar those reported other countries from sub-Saharan [4,26,27], Vietnam [35], and China [38]. The concern in this study is not so much with inequalities per se (important as these are) but rather with the extent to which measured inequality varies according to the weight attached to the poor in the computation of the inequality index. To gain further insight into the nature of this inequality in childhood malnutrition, extended concentration and achievement indices were disintegrated into different sub-groups – geopolitical regions, ethnicity, type of residence, and gender. Many sub-groups that do well on one dimension (e.g. the average) do badly on the other (e.g. inequality). South-east and south-west regions, for example, have low average levels of childhood malnutrition, but the inequalities between the poor and the better-off are very large. The same is true for Igbo and Yoruba ethnic group. By contrast, North-east and North-west have fairly small gaps between the poor and the better-off on childhood malnutrition, but the average values of the childhood malnutrition are extremely high. This study provided evidence that childhood malnutrition is concentrated among women with low education. This has association has been reported in Cameroon[39]. Pongou and colleagues [39] reported that the risk of childhood malnutrition is reduced in educated mothers because they have greater capacity to substitute with less costly sources of nutrients during periods of recessions.

Like other studies have reported, reliance on global averages alone can be misleading [4,40]. Thus, there is a need to take into account inequality as well as the average of health. In assessing achievement it is important to think not just about the mean, nor just about inequality, but about both.

Nigeria is made up of six major geopolitical regions. It is ethnically and religiously diverse and economic development and educational levels vary widely across the country. The North East and North West regions are largely agrarian and predominantly rural. The population level of education is low. The North Central region is one-third urbanized. The South East region is slightly more urbanized than the northern regions. The South West region, which includes Lagos, the former capital is the most urban of the six regions. The South South region is the least urbanized of the three southern regions. Not unexpectedly, this study found that inequality and "disachievement" of childhood malnutrition initiation vary widely by region and ethnic groups. Consistent with findings from Cameroon, there is evidence of regional disparities in childhood malnutrition, such that the childhood malnutrition is worst in the Northern region. Pongou et. al. [7] found Northern Cameroon with dry climate and limited crops had worst weight-for-age. Cameroon shares a long northern-western border with Nigeria. Saharan drought is probable one of the most influential reasons for higher "disachievement" noticed among the Hausas and Northern parts of the country. Rainfall in Nigeria follows a gradient, becoming scarcer the further north one goes and the southern part of the country receives about twice the annual rainfall of the northernmost areas [41]. The pattern of agricultural activity mirrors the rain, with intensive farming concentrated along the southern fridge. Therefore, agricultural activities have been severely affected, and the resulting food security crisis forced people to consume unfit food and polluted water, which in turn affected feeding practices. The lower mean of malnutrition are mainly concentrated in the Western regions. This may be due to regional advantages prior to discovery of oil [42]. People in this states benefited from free education, agricultural settlements, and industrial development. Thus parents are still likely to have more health-care knowledge and enjoy better health conditions, which could effectively lower the prevalence of childhood malnutrition. However, higher level of inequality noticed among Igbos and Hausas and Western part of the country is intriguing and would benefits from further exploration.

Finally, it is necessary to discuss the limitations of this study, as well as its strengths. The present study was performed in a large nationally representative sample with stratified random sampling. This allows for generalizability of the study to whole country. One important limitation is that DHS surveys do not collect data on household income or expenditure, the traditional indicators used to measure wealth. The assets-based wealth index used here is only a proxy indicator for household economic status, and it does not always produce results similar to those obtained from direct measurements of income and expenditure where such data are available or can be collected reliably In addition, the creation of the wealth index was rests on assumption that the underlying variables of the indicator are highly correlated. An alternative method has been developed by Ferguson et. al[43] and implemented in Pongou et. al. [39] and Pongou et. al. [7] Finally, cross-sectional data only allow looking at associations; and does not allow for confident causal inferences.

## Conclusion

There are significant differences in under-five child malnutrition that favour the better-off of society. These are unnecessary, avoidable and unjust. Like other studies have reported, reliance on global averages alone can be misleading. Thus there is a need for evaluating policies not only in terms of improvements in averages, but also improvements in distribution. Addressing problems of stunting, which are found to be responsive to improvements in household income status, requires initiatives that transcend the medical arena. Good nutrition is the cornerstone for survival, health and development. Well-nourished children perform better in school, grow into healthy adults and in turn give their children a better start in life.

## Competing interests

The author declares that they have no competing interests.

## Authors' contributions

OAU conceived the study, extracted data, did the analyses and interpretation, and wrote the first and final draft of the manuscript.
